# Dry eye, sleep quality, and mood status in glaucoma patients receiving prostaglandin monotherapy were comparable with those in non-glaucoma subjects

**DOI:** 10.1371/journal.pone.0188534

**Published:** 2017-11-27

**Authors:** Shugyoku Ra, Masahiko Ayaki, Kenya Yuki, Kazuo Tsubota, Kazuno Negishi

**Affiliations:** 1 Departments of Ophthalmology, Keio University, School of Medicine, Tokyo, Japan; 2 Otake Clinic Moon View Eye Center, Yamato, Japan; Xiamen University, CHINA

## Abstract

**Purpose:**

Prior studies suggested that glaucoma patients suffer worse dry eye and mood and sleep disorders than non-glaucoma subjects. Prostaglandin analogues are first-line therapy for glaucoma, inducing few instillation problems and sufficient pressure-reduction effects. This study compared dry eye, sleep quality, and mood status between glaucoma patients receiving prostaglandin monotherapy and non-glaucoma subjects.

**Methods:**

This cross-sectional study evaluated 1520 patients (579 males and 941 females) for glaucoma status and dry eye-related symptoms (dryness, eye fatigue, photophobia, pain, blurring) and signs (Schirmer test, tear break-up time, corneal staining scores). Of the total cohort, 93 patients were also evaluated by Pittsburgh sleep quality index (PSQI) and hospital anxiety and depression score (HADS). Inclusion criteria were consecutive patients ≥ 51 years of age and best-corrected visual acuity ≥ 20/25. Glaucoma patients included those treated with prostaglandin or a fixed combination including prostaglandin. Exclusion criteria were history of ocular surgery within one month. Data were analyzed using the chi-square or Mann-Whitney U tests, at 5% significance.

**Results:**

There were no significant differences in dry eye-related signs and symptoms between the control (n = 1431, mean age of 66.9 years) and glaucoma groups (n = 89, 67.9 years). The psychiatric sub-analysis of the control (n = 61, 66.2 years) and glaucoma groups (n = 32, 67.3 years) revealed mean scores of 5.02 ± 3.10 and 5.16 ± 3.46 for PSQI (normal range ≤ 5), 9.47 ± 5.61 and 9.42 ± 7.36 for HADS (normal range ≤ 10), 4.84 ± 3.22 and 4.71 ± 3.45 for anxiety (normal range ≤ 5), and 4.63 ± 3.05 and 4.71 ± 4.40 for depression (normal range ≤ 5), respectively, without statistical significance.

**Conclusions:**

Our results were comparable between glaucoma patients on prostaglandin monotherapy and non-glaucoma subjects for dry eye-related clinical manifestations, sleep quality, and mood status.

## Introduction

Topical therapy and laser applications have become major strategies in modern management for glaucoma that strives for less invasive intervention. Following recent advances in topical therapies for glaucoma and its diagnosis, the number of patients using glaucoma eye drops has increased [1] and the use of prostaglandin (PG) analogues and fixed combination therapies in glaucoma treatment is markedly more prominent. All such advances have combined to effectively reduce intraocular pressure, which is beneficial for both patients and ophthalmologists.

Ocular surface side effects are mostly from preservatives contained in glaucoma eye drops [2,3]. Patients may feel some discomfort during the use of such eye drops including irritation, burning, stinging, and a foreign body sensation.[4] Benzalkoniun chloride (BAK) is the primary preservative used in glaucoma eye drops [5,6], while other preservatives are also used to reduce toxicity including SofZia^TM^ (Alcon Laboratories, Tokyo, Japan) and PolyQuad^TM^ (Alcon Laboratories, Tokyo, Japan).Numerous reports detail BAK cytotoxicity [7,8,9], which generally depends on BAK concentrations.

Recent studies suggested that glaucoma patients tend to have sleep disturbance [10–12] and mental disorder, while other groups reported glaucoma patients were depressive in comparison to non-glaucoma subjects [13–16], although the severity of glaucoma and visual field defects may contribute to the depression. Damage of intrinsically photosensitive retinal ganglion cells (ipRGCs) was proposed as underlying condition in sleep disorder of glaucoma patients [11] and the presence of circadian rhythm disorder has also been suggested to associate with glaucoma, [17], while the burden of eyedrop instillation might also contribute to sleep disturbance [12].

PGs have several benefits for pressure reduction and prevention of side effects since currently introduced PGs contain only a small amount of BAK and require a single instillation per day. Compared to conventional therapies with multiple medication and instillation, a single instillation is free from cumulative toxicity to ocular surface [18] and the burden of daily medication management [12]. Consequently, patient’s adherence was significantly improved with accompanying substantial reductions in intraocular pressure [19–21]. Taken together, previous findings indicated that glaucoma patients receiving PG monotherapy are expected to have less negative effects with regard to ocular and psychiatric health. However, to the best of our knowledge, there are few investigations comparing sleep status between non-glaucoma subjects and glaucoma patients taking PGs.

The aim of this study was to compare ocular surface complications, sleep quality, and mood status between glaucoma patients on prostaglandin monotherapy and non-glaucoma subjects. We conducted a multi-center study in a large cohort under the standardized diagnostic criteria for glaucoma and dry eye disease (DED) to minimize selection bias.

## Methods

### Study institutions and Institutional Review Board approval

The Institutional Review Boards and Ethics Committees of Keio University School of Medicine, Shinseikai Toyama Hospital, Shinkokai Medical Group, and Komoro Kosei Hospital approved this study, and the study was performed in accordance with the principles of the Declaration of Helsinki. Informed consent was obtained from all participants.

The study was performed in Shinseikai Toyama Hospital (Imizu, Japan), Todoroki Eye Clinic (Tokyo, Japan), Wakita Eye Clinic (Tokyo, Japan), Jiyugaoka Ekimae Eye Clinic (Tokyo, Japan), Tsukuba Central Hospital (Ibaragi, Japan), Takahashi Hisashi Eye Hospital (Akita, Japan), and Ayame Clinic (Saitama, Japan). Study participants, both glaucoma patients and normal controls, were consecutively recruited from patients attending the eye clinics from January 2014 to May 2016.

### Ophthalmological examinations and treatments

Board-certified ophthalmologists examined all patients, with diagnostic criteria for glaucoma in the present study comprising glaucomatous visual field loss less than –4.0 dB MD in the worse eye, an ophthalmoscopic NFL defect, a c/d ratio > 0.6, or elevated IOP (> 21 mmHg) requiring topical medication for more than 6 months. Exclusion criteria were coexisting cataract with significant lens opacity disturbing the optical axis that accounted for subjective visual disturbance or decreased visual function, retinal pathology, retinal surgery, or photocoagulation affecting the visual field. Topical glaucoma medication included Xalatan^®^ (Pfizer, Tokyo, Japan), Tapros^®^ (Santen Pharmaceutical Co. Ltd., Osaka, Japan), Travatanz^®^ (Alcon Laboratories, Tokyo, Japan), Lumigan^®^ (Senjyu Pharmaceutical Co. Ltd., Osaka, Japan), and Xalacom^®^ (Pfizer, Tokyo, Japan). Table 1 (S1 Table) describes the distribution of prescribed PGs. The concentration of BAK in the medications was 0.001% to 0.02%. The low-toxicity preservatives, PolyQuald^®^ is included in Travatanz^®^.

**Table 1:** Distribution of prescribed prostaglandin analogues among glaucoma patients in the sleep study.

Trade name	Dry eye study group (n)	Sleep study group (n)	Preservatives
Xalatan®	60	18	BAK 0.02%
Tapros®	15	6	BAK 0.001%
Travatanz®	12	6	SofZia®
Lumigan®	2	1	BAK 0.005%

BAK, benzalkonium chloride

A diagnosis of DED was made according to the Japanese Dry Eye Society,[22] which classifies DED into definite DED (DDED), probable DED (PDED), and non-DED based on the presence of dry eye symptoms, tear abnormalities (Schirmer test ≤5 mm or tear BUT ≤5 s), and superficial punctate keratoepitheliopathy (staining score ≥3). Patients diagnosed with DDED or PDED were enrolled in the present study as subjects with DED. None of the patients had undergone any non-medical interventions, such as punctal plug insertion or punctal occlusion, or any surgical interventions. The eye drops prescribed for the treatment of DED contained hyaluronate, mucin secretagogue, and steroid, i.e., 0.1%/0.3% Hyalein^R^ (sodium hyaluronate; Santen Pharmaceutical Co. Ltd., Osaka, Japan) with 0.003%BAK, 0.1%/0.3% Tearbalance^R^ (sodium hyaluronate; Senju Pharmaceutical Co. Ltd., Osaka, Japan) with no BAK, Diquas^R^ (3% diquafosol sodium; Santen Pharmaceutical Co. Ltd) with no BAK, Mucosta^R^ (2% rebamipide, Otsuka Pharmaceutical, Co. Ltd., Tokyo, Japan) with no BAK, and 0.02%/0.1% Flumetholon^R^ (fluorometholon; Santen Pharmaceutical Co. Ltd) with 0.005%BAK. Other topical medications included Kary® Uni containing 0.005%pinorexine (Santen Pharmaceutical Co. Ltd) with 0.005%BAK and Sancoba® containing 0.02%cyanocobaramine (Santen Pharmaceutical Co. Ltd) with 0.005%BAK.

### Patient interviews for DED-related symptoms

Outpatients were interviewed regarding symptoms related to DED. Questions determined the presence or absence of six common DED-related symptoms, namely dryness, irritation, pain, eye fatigue, blurring, and photophobia. The questions put to patients were selected from items in the Dry Eye Questionnaire Score (DEQS)[23], which is based on the six most prevalent symptoms of DED patients visiting the Dry Eye Clinic of the Department of Ophthalmology, Keio University Hospital, in 2012.

### Questionnaire-based survey for sleep and mood disorders

Outpatients were invited to complete the Pittsburgh Sleep Quality Index (PSQI)[24] and the Hospital Anxiety and Depression Scale (HADS)[25] questionnaires. Scores for each scale were calculated according to separate algorithms and then subjected to analysis. The cut-off point for PSQI was a total score of 5 or greater, being indicative of poor sleep quality. The cut-off points for the HADS were 0–10 as normal and 11–21 as abnormal. The PSQI consists of seven subscales, whereas the HADS consists of depression (HADS-D) and anxiety (HADS-A) subscales. These questionnaires have been widely used for hospital-based surveys and are easy to answer, even by eye clinic visitors, because they do not contain difficult questions concerning severe psychiatric disease (e.g. suicide and hallucinations).

### Patient evaluations for systemic comorbidities

The presence of systemic comorbidities was evaluated on the basis of brief interviews, chart review, and results from annual health check records, if available. Assessments of the use of sleep medications and antidepressants were included in the PSQI score.

### Inclusion and exclusion criteria for people with eye disease and systemic comorbidities

The present study recruited consecutive patients older than 50 years with best corrected visual acuity equal to or better than 20/25 in both eyes, from participating clinics during the study period. In addition, patients with ocular surgery within one month, diagnosed psychiatric disease, and taking specific psychiatric medications were excluded from the study. DED is often accompanied with sleep and mood disorders, and therefore those with DED were excluded from the sleep study [26] so as to not overestimate sleep and mood status.

### Statistical analysis

Subjects were divided into two groups: glaucoma patients treated with PG-containing eyedrops and non-glaucoma subjects. Results for DED-related signs and symptoms as well as sleep and mood disorders were compared between groups, based on results of the Schirmer test and the tear BUT in the right eye.

Where appropriate, data are given as the mean ± SD. Chi-square and Mann-Whitney U tests were used as appropriate for all analyses, which were performed using StatFlex (Atech, Osaka, Japan) with *P* < 0.05 considered significant.

## Results

We evaluated 1520 patients (579 males and 941 females) by comprehensive glaucoma and dry eye examinations. A total of 93 patients in this series were also evaluated for sleep quality and mood status using two questionnaires.

**Fig 1:**
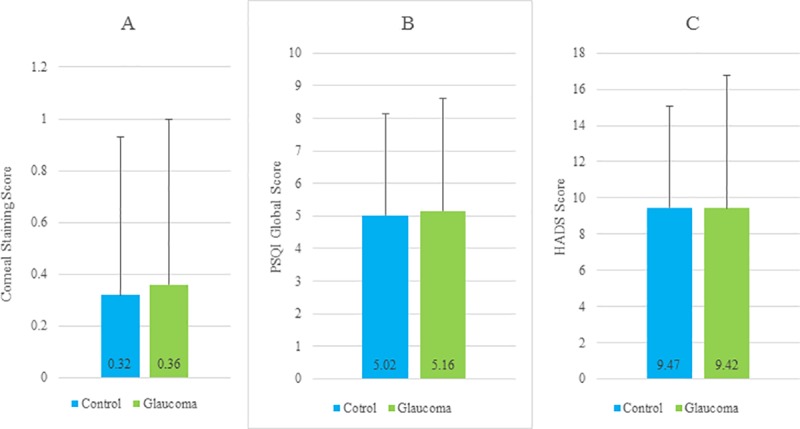
Comparison of representative parameters for dry eye disease, sleep, and mood between an affected and control groups. There was no significant difference between the control patients those with glaucoma in corneal staining score (Fig 1A), PSQI global score (Fig 1B), and HADS score (Fig 1C). The data are given as mean ± SD. PSQI, Pittsburgh Sleep Quality Index; HADS, Hospital Anxiety and Depression Scale; HADS-A, Anxiety subscale; HADS-D, Depression subscale.

Patient demographics and univariate comparisons of clinical parameters are given in Tables 2 and 3 (S1 and S2 Tables), and indicated no significant difference in either DED-related symptoms and signs or sleep and mood indices between the glaucoma and control groups. Dry eye related eyedrops and other topical medications were not different between two groups (Table 2). Comparisons of representative data between the groups are shown in Fig 1A, 1B and 1C which reveals no significant difference between the groups in corneal staining score, PSQI global score, and HADS score.

**Table 2:** [Dry eye study] Patient demographics and univariate comparisons of clinical parameters.

	Control	Glaucoma	*P*-value ^A^
No. subjects	1431	89	
Age (years)	66.9 ± 9.7	67.9 ± 9.2	n.s.
No. males/females	534/897	45/44	0.013*
Dry eye-related symptoms			
Dryness +/-	345/1086	19/70	n.s.
Eye fatigue +/-	456/975	25/64	n.s.
Photophobia +/-	247/1176	11/78	n.s.
Pain +/-	93/1333	5/84	n.s.
Blurring +/-	273/1151	18/71	n.s.
Dry eye-related signs			
Schirmer test (≤ 5 mm/> 5mm)	46/134	2/8	n.s.
Tear break-up time (≤ 5s/> 5s)	700/726	49/39	n.s.
Corneal staining score	0.32 ± 0.61	0.36 ± 0.64	n.s.
Topical medications other than prostaglandin (% of cases)			
None	62.5	53.8	0.866
Sodium hyarulonate	12.4	22.0	0.127
Mucin secretagogue	12.2	13.2	
Steroid + Hyarulonate	3.1	0.0	
Others^B^	9.8	11.0	0.702

Unless indicated otherwise, data are given as the mean ± SD.

^A^*P*-values were obtained by Chi-squared test and the Mann–Whitney U test, as appropriate; asterisks indicate significant differences.

^B^other medications included Kary® Uni and Sancoba®.

**Table 3:** [Sleep study] Patient demographics and univariate comparisons of clinical parameters.

	Control	Glaucoma	*P*-value^A^
No. subjects	61	32	
Age (years)	66.2 ± 8.2	67.3 ± 8.0	n.s.
No. males/females	24/37	9/23	n.s.
Visual acuity and IOP			
LogMAR in the worse eye	–0.03 ± 0.05	0.07 ± 0.17	0.000*
LogMAR in the better eye	–0.05 ± 0.04	0.01 ± 0.11	0.001*
IOP (mmHg)	14.0 ± 5.2	15.5 ± 4.4	n.s.
Visual field			
MD in the worse eye (dB)	–1.85 ± 3.94	–6.90 ±7.82	0.001*
MD in the better eye (dB)	-0.46 ± 2.32	–1.53 ± 3.37	n.s.
Sleep and mood indices			
Sleep parameters			
PSQI global score	5.02 ± 3.10	5.16 ± 3.46	n.s.
Sleep latency (h)	0.46 ± 0.90	0.36 ± 0.41	n.s.
Sleep efficacy (%)	91.8 ± 17.3	95.4 ± 13.5	n.s.
Mood parameters			
HADS score	9.47 ± 5.61	9.42 ± 7.36	n.s.
HADS-A subscore	4.84 ± 3.22	4.71 ± 3.45	n.s.
HADS-D subscore	4.63 ± 3.05	4.71 ± 4.40	n.s.

Unless indicated otherwise, data are given as the mean ± SD.

^A^*P*-values were obtained by Chi-squared test and the Mann–Whitney U test, as appropriate; asterisks indicate significant differences.

IOP, intraocular pressure in the worse eye; MD, mean deviation of Humphrey Field Analyzer 30–2.

PSQI, Pittsburgh Sleep Quality Index; HADS, Hospital Anxiety and Depression Scale; HADS-A, Anxiety subscale; HADS-D, Depression subscale.

## Discussion

Our results demonstrated that PG monotherapy had no negative impacts on ocular surface health, sleep, and mood in glaucoma patients. Despite that DED patients often report discrepancies between symptoms and signs [27], and no examined parameters showed inconsistency as to whether glaucoma patients receiving PG monotherapy really enjoy reduced ocular discomfort. This benefit should contribute to better adherence and avoidance of additional care for ocular surface management. Recent formulation of glaucoma eye drops contains less or no BAK and it may be the cause of the improved tolerance resulting in decreased side effects.

There was no difference in the sleep and mood indices between the glaucoma and control patients, while previous reports showed that glaucoma patients tended to have sleep and mood disorders [10–16]. The reason for this apparent discrepancy is that the present study may not include very severe cases since all patients were controlled with PG monotherapy only and the burden of medication was minimal. Previous investigations with detailed ophthalmological evaluation included many cases with severe visual field loss and multiple medications and they indicated the psychiatric disorders were associated with the severity of glaucoma [10–13].

Damage to the ipRGC is an attractive hypothesis for sleep disturbance in glaucoma although clinical evidence to support this proposal are still to be determined. Gracitelli et al demonstrated decreased sleep quality in glaucoma patients with electroencephalography and it was correlated to reduced ipRGC function measured with a pupillometer under blue-light stimulus [11]. Actually, numerous factors affect sleep quality in aged glaucoma patients and we speculate many glaucoma patients may be depressive due to the progressive nature of the disease and the burden of life-long medication use [12], thus it is likely that depression may be a major cause of sleep disorders in such patients since it is strongly correlated with sleep disturbance [28,29].

Social awareness of glaucoma and advancement of optical coherence tomography have led to easy and early diagnosis of glaucoma [30], and many patients are now treated appropriately. A recent review of PG therapy describes no significant side effects for the ocular surface [31]. We therefore speculate that cumulative epidemiological evidence, diagnostic and pharmaceutical advancements, and reduced preservative in PGs may contribute to both effective treatment and prevention of dry eye and psychiatric disorders in glaucoma patients receiving PG monotherapy.

### Limitations

The present study has limitations. The gender ratio between control and glaucoma groups in dry eye study were significantly different, however, we believe it did not affect the results since the difference may not be clinically significant and topical medications were similar in both groups. For more informative results, sleep and mood in such patients should be further evaluated with polysomonography (electroencephalogram) and psychiatric consultation. In addition, further investigations should be conducted in patients with severe glaucoma being treated with beta-blocker eyedrops with a single instillation formulation. Finally, the number of subjects who completed the Schirmer test was small, although still acceptable because it was not among the essential new diagnostic criteria for dry eye [32].

## Conclusions

In conclusion, our results demonstrated no significant difference between control and glaucoma groups in DED, sleep, and mood, suggesting that adverse ocular and psychiatric effects with prostaglandin monotherapy might be minimal.

## Supporting information

S1 Table (XLS)

S2 Table (XLS)

## References

[1] Weinreb RN, Khaw PT. Primary open-angle glaucoma. Lancet. 2004;363:1711–1720. doi: 10.1016/S0140-6736(04)16257-0 15158634 10.1016/S0140-6736(04)16257-0

[2] Baudouin C, Labbé A, Liang H, Pauly A, Brignole-Baudouin F. Preservatives in eyedrops: the good, the bad and the ugly. Prog Retin Eye Res. 2010 7;29(4):312–34. doi: 10.1016/j.preteyeres.2010.03.001 20302969 10.1016/j.preteyeres.2010.03.001

[3] Ramli N, Supramaniam G, Samsudin A, Juana A, Zahari M, Choo MM. Ocular Surface Disease in Glaucoma: Effect of Polypharmacy and Preservatives. Optom Vis Sci. 2015 9;92(9):e222–6. doi: 10.1097/OPX.0000000000000542 25730335 10.1097/OPX.0000000000000542

[4] Fukuchi T, Wakai K, Suda K, et al Incidence, severity and factors related to drug-induced keratoepitheliopathy with glaucoma medications. Clin Ophthalmol 2010;26:203–209. 10.2147/opth.s9716 PMC2861924 20463785

[5] Baudouin C, Renard JP, Nordmann JP, Denis P, Lachkar Y, Sellem E, et al Prevalence and risk factors for ocular surface disease among patients treated over the long term for glaucoma or ocular hypertension. Eur J Ophthalmol. 2012 6 11:0 doi: 10.5301/ejo.5000181 22729444 10.5301/ejo.5000181

[6] Uematsu M, Kumagami T, Shimoda K, Kusano M, Teshima M, Sasaki H, et al Influence of Alkyl Chain Length of Benzalkonium Chloride on Acute Corneal Epithelial Toxicity. Cornea. 2010 11;29(11):1296–301. doi: 10.1097/ICO.0b013e3181dc81b6 20802315 10.1097/ICO.0b013e3181dc81b6

[7] Furrer P, Mayer JM, Gurny R. Ocular tolerance of preservatives and alternatives. Eur J Pharm Biopharm. 2002 5;53(3):263–80. Review. 11976014 10.1016/s0939-6411(01)00246-6

[8] Iwasawa A, Ayaki M, Niwano Y. Cell viability score (CVS) as a good indicator of critical concentration of benzalkonium chloride for toxicity in cultured ocular surface cell lines. Regul Toxicol Pharmacol. 2013 7;66(2):177–83. doi: 10.1016/j.yrtph.2013.03.014 Epub 2013 Apr 1. 23557985 10.1016/j.yrtph.2013.03.014

[9] Ayaki M, Iwasawa A, Niwano Y. Preservatives in glaucoma medication. Leading article in Review Section. CML Ophthalmology 2013;23(2):45–52.

[10] Agorastos A, Skevas C, Matthaei M, Otte C, Klemm M, Richard G, et al Depression, anxiety, and disturbed sleep in glaucoma. J Neuropsychiatry Clin Neurosci. 2013 Summer;25(3):205–13. doi: 10.1176/appi.neuropsych.12020030 24026713 10.1176/appi.neuropsych.12020030

[11] Gracitelli CP, Duque-Chica GL, Roizenblatt M, Moura AL, Nagy BV, Ragot de Melo G, et al Intrinsically photosensitive retinal ganglion cell activity is associated with decreased sleep quality in patients with glaucoma. Ophthalmology. 2015 6;122(6):1139–48. doi: 10.1016/j.ophtha.2015.02.030 25858174 10.1016/j.ophtha.2015.02.030

[12] Ayaki M, Shiba D, Negishi K, Tsubota K. Depressed visual field and mood are associated with sleep disorder in glaucoma patients. Sci Rep. 2016 5 11;6:25699 doi: 10.1038/srep25699 27168309 10.1038/srep25699 PMC4863426

[13] Mabuchi F, Yoshimura K, Kashiwagi K, Yamagata Z, Kanba S, Iijima H, et al Risk factors for anxiety and depression in patients with glaucoma. Br J Ophthalmol. 2012 6;96(6):821–5. doi: 10.1136/bjophthalmol-2011-300910 22353697 10.1136/bjophthalmol-2011-300910

[14] Jung KI, Park CK. Mental Health Status and Quality of Life in Undiagnosed Glaucoma Patients: A Nationwide Population-Based Study. Medicine (Baltimore). 2016 5;95(19):e3523 doi: 10.1097/MD.0000000000003523 27175648 10.1097/MD.0000000000003523 PMC4902490

[15] Kong X, Yan M, Sun X, Xiao Z. Anxiety and depression are More Prevalent in Primary Angle Closure glaucoma Than in Primary Open-Angle glaucoma. J Glaucoma 2015; 24: e57–63. doi: 10.1097/IJG.0000000000000025 24240874 10.1097/IJG.0000000000000025

[16] Kong XM, Zhu WQ, Hong JX, Sun XH. Is glaucoma comprehension associated with psychological disturbance and vision-related quality of life for patients with glaucoma? A cross-sectional study. BMJ Open 2014; 4: e004632 doi: 10.1136/bmjopen-2013-004632 24861547 10.1136/bmjopen-2013-004632 PMC4039808

[17] Wang H, Zhang Y, Ding J, Wang N. Changes in the circadian rhythm in patients with primary glaucoma. PLoS One. 2013 4 29; 8(4):e62841 doi: 10.1371/journal.pone.0062841 23658653 10.1371/journal.pone.0062841 PMC3639222

[18] Ayaki M, Iwasawa A, Niwano Y. Cell viability score as an integrated indicator for cytotoxicity of benzalkonium chloride-containing antiglaucoma eyedrops. Biocontrol Sci. 2012;17(3):121–8. 23007103 10.4265/bio.17.121

[19] Tsai JC, McClure CA, Ramos SE, Schlundt DG, Pichert JW. Compliance barriers in glaucoma: a systematic classification. J Glaucoma. 2003 10;12(5):393–8. 14520147 10.1097/00061198-200310000-00001

[20] Schwartz GF, Quigley HA. Adherence and persistence with glaucoma therapy. Surv Ophthalmol. 2008 11;53 Suppl1:S57–68. doi: 10.1016/j.survophthal.2008.08.002 Review. 19038625 10.1016/j.survophthal.2008.08.002

[21] Robin AL, Novack GD, Covert DW, Crockett RS, Marcic TS. Adherence in glaucoma: objective measurements of once-daily and adjunctive medication use. Am J Ophthalmol. 2007 10;144(4):533–40. doi: 10.1016/j.ajo.2007.06.012 17686450 10.1016/j.ajo.2007.06.012

[22] Shimazaki J, Tsubota K, Kinoshita S, Ohashi Y. Definition and diagnosis of dry eye 2006. Atarashii Ganka 2007; 24:181–184. In Japanese.

[23] Sakane Y, Yamaguchi M, Yokoi N, Uchino M, Dogru M, Oishi T, et al Development and validation of the Dry Eye-Related Quality-of-Life Score questionnaire. JAMA Ophthalmol 2013;131:1331–1338. doi: 10.1001/jamaophthalmol.2013.4503 23949096 10.1001/jamaophthalmol.2013.4503

[24] Buysse DJ, Reynolds CF 3rd, Monk TH, Berman SR, Kupfer DJ. The pittsburgh sleep quality index: A new instrument for psychiatric practice and research. Psychiatr Res 1989;28:193–213. 10.1016/0165-1781(89)90047-4 2748771

[25] Zigmond AS, Snaith RP. The hospital anxiety and depression scale. Acta Psychiatr Scand. 1983;67:361–370. 6880820 10.1111/j.1600-0447.1983.tb09716.x

[26] Ayaki M, Kawashima M, Negishi K, Kishimoto T, Mimura M, Tsubota K. Sleep and mood disorders in women with dry eye disease. Sci Rep. 2016 10 12;6:35276 doi: 10.1038/srep35276 27731398 10.1038/srep35276 PMC5059662

[27] Schmidl D, Witkowska KJ, Kaya S, Baar C, Faatz H, Nepp J, et al The association between subjective and objective parameters for the assessment of dry-eye syndrome. Invest Ophthalmol Vis Sci. 2015;56: 1467–1472. doi: 10.1167/iovs.14-15814 25650419 10.1167/iovs.14-15814

[28] Breslau N, Roth T, Rosenthal L, Andreski P. Sleep disturbance and psychiatric disorders: a longitudinal epidemiological study of young adults. Biol Psychiatry. 1996 3 15;39(6):411–8. 8679786 10.1016/0006-3223(95)00188-3

[29] Nutt D, Wilson S, Paterson L. Sleep disorders as core symptoms of depression. Dialogues Clin Neurosci. 2008;10(3):329–36. Review. 18979946 10.31887/DCNS.2008.10.3/dnutt PMC3181883

[30] Jung Y, Park HY, Jeong HJ, Choi SY, Park CK. The Ability of 10–2 Short-Wavelength Perimetry in Detecting Functional Loss of the Macular Area in Preperimetric Glaucoma Patients. Invest Ophthalmol Vis Sci. 2015 12;56(13):7708–14. doi: 10.1167/iovs.15-17819 26641548 10.1167/iovs.15-17819

[31] Aihara M. Prostaglandin analogues. Program for professional education in Ophthalmology. A review 70. J Jpn Ophthalmol Soc. 2017;117: 389–400. Review (in Japanese)

[32] Tsubota K, Yokoi N, Shimazaki J, Watanabe H, Dogru M, Yamada M, et al New Perspectives on Dry Eye Definition and Diagnosis: A Consensus Report by the Asia Dry Eye Society. Ocul Surf 2017; 15:65–76. doi: 10.1016/j.jtos.2016.09.003 Review. 27725302 10.1016/j.jtos.2016.09.003

